# ‘Hierarchy’ in the organization of brain networks

**DOI:** 10.1098/rstb.2019.0319

**Published:** 2020-02-24

**Authors:** Claus C. Hilgetag, Alexandros Goulas

**Affiliations:** 1 Institute of Computational Neuroscience, University Medical Center Eppendorf, Hamburg University, Hamburg, Germany; 2 Department of Health Sciences, Boston University, Boston, MA, USA

**Keywords:** visual cortical hierarchy, laminar projection patterns, concerted cortical gradients, spatial temporal scales

## Abstract

Concepts shape the interpretation of facts. One of the most popular concepts in systems neuroscience is that of ‘hierarchy’. However, this concept has been interpreted in many different ways, which are not well aligned. This observation suggests that the concept is ill defined. Using the example of the organization of the primate visual cortical system, we explore several contexts in which ‘hierarchy’ is currently used in the description of brain networks. We distinguish at least four different uses, specifically, ‘hierarchy’ as a topological sequence of projections, as a gradient of features, as a progression of scales, or as a sorting of laminar projection patterns. We discuss the interpretation and functional implications of the different notions of ‘hierarchy’ in these contexts and suggest that more specific terms than ‘hierarchy’ should be used for a deeper understanding of the different dimensions of the organization of brain networks.

This article is part of the theme issue ‘Unifying the essential concepts of biological networks: biological insights and philosophical foundations’.

## 1. Introduction

### (a) Background

*Hierarchy* is one of the most popular terms in current network and systems neuroscience.^1^ A combined pubmed (pubmed.gov) search of the keywords ‘hierarchy’ and ‘brain’ and ‘network’ yields more than 2500 references, with strongly increasing frequency over the last 15 years. However, the sense in which ‘hierarchy’ is used in these publications can vary from one paper to the next, or even within the same paper. For example, when addressing ‘…the hierarchical arrangement of cortical sensory areas’, a study [1] may refer to concepts of laminar-specific projections [2], topological projection sequence [3], as well as a combination of both [4]. Similarly, descriptions of how ‘hierarchy’ is expressed in the human and non-human primate brain [5] may interchangeably employ different perspectives of ‘hierarchy’, such as distance along the posterior-anterior axis of the brain, ordered variations of neural responses in terms of functional complexity, gradients of cortical thickness, or a progression of laminar projection patterns. These are clearly very different matters, and while many neuroscientists have the intuitive feeling that these notions of ‘hierarchy’ are somehow related, it is impossible to establish whether this is true or not and in which way the different interpretations may be linked without carefully exploring each of the different ‘hierarchy’ concepts in turn. Failure to do so is bound to result in confusion.

### (b) The example of the visual cortical hierarchy

**Figure 1.**
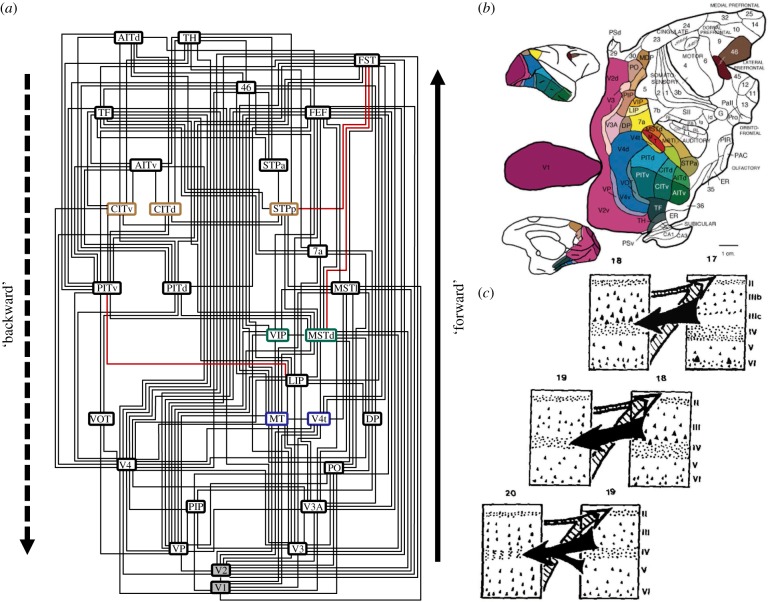
Visual cortical hierarchy (VCH) based on the sorting of cortico-cortical projections according to their direction inferred from the laminar patterns of projection origins and terminations. (*a*) Example of an optimal arrangement of visual cortical areas of the primate (macaque) cerebral cortex, according to the sorting of oriented projections, where the orientation was inferred from the laminar patterns of projection origins and terminations [6]. In this framework, projections that originate predominantly from the upper, supergranular cortical layers are considered to be ‘forward’ projections (which are arranged to point up), while projections that originate predominantly from the deep, infragranular cortical layers are considered to be ‘backward’ projections (arranged to point down), following the convention of [2]. Generally, connections in the diagram reflect reciprocal relations of laminar projections. The three red connections indicate the minimal set of (six) laminar relations that are violated in the overall arrangement. Areas with boxes of the same colour maintain their relative level positions across all optimal arrangements, while areas with a shaded background (i.e. V1 and V2) are the only areas to stay fixed on the first and second level, respectively. For more details see [6]. (*b*) Map of the macaque visual cortex with areas forming part of the VCH shown in colour [2]. (*c*) The notion of a direction of projections that is associated with laminar patterns was derived from the observation that laminar projection origins and terminations show highly regular, repeating patterns as one proceeds from areas at the sensory periphery (A17, striate cortex, primary visual cortex) to more central areas of the brain (e.g. A18, A19) [7]. (Online version in colour.)

Let us consider one traditional notion of ‘hierarchy’ in brain networks in more detail. A classic interpretation of ‘hierarchy’ that represents to many systems neuroscientists a fundamental version of the concept is the primate visual cortical hierarchy (VCH). This concept is enshrined in the wiring diagram of Felleman & Van Essen [2] (figure 1*a* shows an updated version of the diagram). This diagram has been presented at a large number of neuroscience meetings, to impress on the audience the complexity of the wiring of the cerebral cortex, combined with a comforting approach for restoring order. If shown without further explanation, the diagram is frequently misunderstood by a part of the audience which assumes that the areas are arranged according to how many synaptic stages they are removed from direct visual input. Moreover, it is frequently expected that response properties change systematically as one moves to higher-level areas within the scheme. In particular, receptive fields should become larger and the topographic (retinotopic) organization less pronounced, so that neurons at higher levels of the scheme respond to more global and complex image features, such as faces and complex motion features.

**Figure 2.**
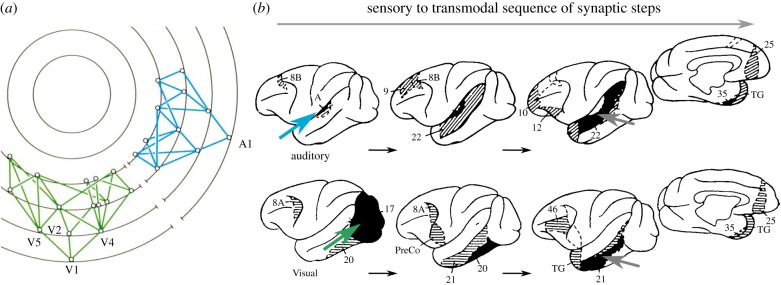
Topological arrangement of sensory (visual and auditory) connections in the primate cortex. (*a*) Connections of visual cortical areas in the macaque brain, arranged by topological distance. Each concentric ring represents a different synaptic level, starting with primary sensory cortex on the outermost level 1. Any two levels are separated by at least one unit of synaptic distance. Nodes at the same level are reciprocally interconnected by the black arcs of the concentric rings. Figure and description adapted from [3]. (*b*) Progression of connections from the primary visual (green arrow) and auditory (blue arrow) areas of the primate cortex. Each new step is shown in black and the further connections of the new areas by light stippling or hatching. All sensory pathways converge in the depths of the superior temporal sulcus (STS, grey arrow). Adapted from [8].

In fact, the areas are not arranged by topology, that is, synaptic step distance from the retina (as they are in the diagram by Mesulam [3] shown in figure 2*a*), but according to the laminar origin and termination patterns of projections between them. Projections that originate mostly from supragranular (upper) cortical layers and terminate in the granular layer (layer IV) are classified as ‘forward’, while projections that originate mostly in infragranular (deep) cortical layers and terminate outside the granular layer and particularly in the upper cortical layers are classified as ‘backward’ [7] (figure 1*c*). The classification can be further extended and refined; specifically, by including categories for ‘lateral’ projections (which originate from upper and deep cortical layers in a more equal proportion and terminate in the target areas in a column-like fashion across all layers) [2].^2^ In order to build the VCH diagram, areas are arranged in such a way that as many as possible ‘forward’ projections point up, while as many as possible ‘backward’ projections point down. In addition, ‘lateral’ connections link areas at the same level. In fact, many (hundreds of thousands) different schemes can be built that all fulfill these constraints optimally, with a minimum number of (six) constraint violations [6,9]. Therefore, strictly speaking, there exists not a single VCH, but many different ones that vary substantially by the number of levels and the sequence of areas (figure 1*a* shows an average optimal scheme). Thus, the VCH is fundamentally indeterminate, even though suggestions have been made for constraining the scheme with the help of quantitative laminar projection patterns [10,11] or by effectively averaging the different schemes [12]. The indeterminacy needs to be taken into account if the VCH diagram is to be interpreted directly as a flowchart of visual signal processing [13].

It is nonetheless interesting that this sorting of laminar-specific projections can be achieved nearly perfectly, with a very small number of violations, on the direction of the more than 300 projections. In addition, most of these violations may be plausibly resolved [6]. This observation indicates an amazing degree of regularity of the laminar cortical projection data. We return to this subject in §5 of the present paper. In any case, it is clear that the iconic wiring diagram of Felleman & Van Essen [2] does not represent topological distance from the retina as is often assumed, but the complicated outcome of a sorting process of cortical areas based on the directional classification of inter-areal projections which is, in turn, based on their laminar origin and termination patterns. The functional implications of this high-order scheme are not obvious, even if ignoring the principal indeterminacy of the sorting process.

Given that the scheme differs fundamentally from how it is frequently understood, it should be no surprise that the VCH has not fulfilled a number of functional expectations invested into it, such as the alignment with systematic changes in response features described above. Specifically, it was found that the VCH scheme does not align well with functional properties of the visual system such as stimulus processing latencies, which are much more simultaneous than expected, in particular in the dorsal part of the visual system [14,15], or the presumed increasing complexity of functional responses of areas on different levels of the VCH [16–18]. These findings were summed up by Zeki [19, p. 243] in the conclusion that ‘… perceptual hierarchy cannot be predicted from the anatomical hierarchy’ (i.e. the VCH), echoing the statement of Silvanto [20, p. 17] that ‘… anatomical hierarchy does not imply functional hierarchy’.

The apparent failure of the VCH scheme to be helpful for a functional interpretation of cortical connectivity was also pointedly summarized by Hegdé & Felleman [21, p. 416]

‘…a growing body of evidence, including recent direct experimental comparisons of functional properties at two or more levels of the anatomical hierarchy, indicates that visual processing neither is hierarchical nor parallels the anatomical hierarchy. Recent results also indicate that some of the pathways of visual information flow are not hierarchical, so that the anatomical hierarchy cannot be taken as a strict flowchart of visual information either. Thus, while the sustaining strength of the notion of hierarchical processing may be that it is rather simple, its fatal flaw is that it is overly simplistic’.

This conclusion may be largely owing to a misunderstanding of the kind of ‘hierarchy’ reflected in the VCH. We revisit the VCH and the question of how laminar patterns of projection origins and terminations can give rise to meaningful functional interpretations in §5 of this paper.

### (c) The plan of the present paper

In this review, we explore different senses of ‘hierarchy’, as they are currently employed in systems neuroscience, such as in the meaning of a topological sequence of projections, as sequences or gradients of structural or functional cortical features and finally the interpretation of ‘hierarchy’ as an ordering of scales of connectivity, as in the case of multi-level modular networks. This exploration might help to differentiate the different senses of ‘hierarchy’. We also attempt to sketch out specific functional implications of the different meanings of ‘hierarchy’. In this way, we hope to delineate and clarify the different concepts of ‘hierarchy’ and demonstrate their functional implications and value.

## 2. Hierarchy as a topological projection sequence

**Figure 3.**
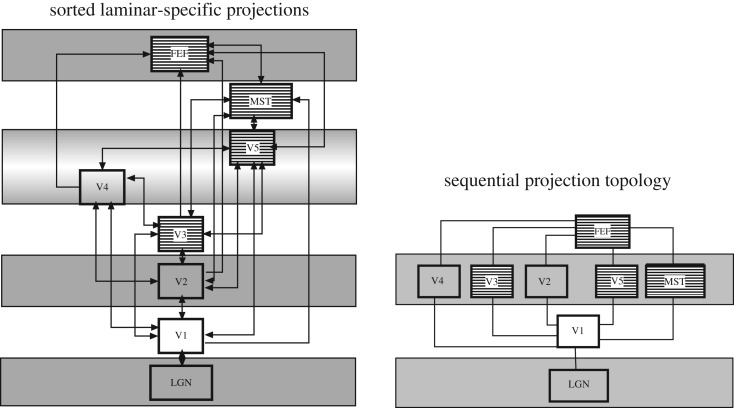
Visual cortical hierarchy versus topological sequence of primate visual cortical projections. The VCH diagram on the left was derived by optimization analysis of the laminar connectivity data between these structures using methods described previously [6]. Areas with near-simultaneous onset latencies are shaded. It is apparent that the stations with near-simultaneous onsets are on different levels of the hierarchy. An optimal topological diagram for the same network, on the right, derived by analysis of the shortest path to each structure from the retina. Stations with near-simultaneous onset latencies are shaded. It is apparent that the stations with near-simultaneous onsets are on the same level of the topological sequence, with the sole exception of the frontal eye fields (FEF). From [22].

We start by taking at face value the intuitive interpretation of the VCH as a topological sequence of projections. In this perspective, primate visual cortical areas are ordered by the number of steps by which they can be reached from sensory (specifically, visual) input, taking the shortest path via direct structural connections (cf. figure 2). Simply, this perspective only considers the direct area-to-area connections, but not their laminar patterns. Directionality in this kind of hierarchy arises from defining a start and end point, such as sensory input or motor output. If primate visual cortical areas are ordered by this sequential connectional topology, rather than by their direction as inferred from laminar patterns, two very different arrangements emerge (figure 3). Conspicuously, the arrangement by the number of connection steps from the retina results in a much more compressed scheme than the VCH. Intuitively, as the scheme takes into account the number of processing steps, it should correlate more closely with functional properties of the visual system, such as onset latencies, than the VCH. Indeed, computational modelling by populations of integrate-and-fire neurons has demonstrated that empirical onset latencies in the primate visual system can be reproduced nearly perfectly on the basis of the connectional topology of the cortical network, without attributing special roles to ‘forward’ and ‘backward’ projections [22]. Remaining discrepancies may point to additional direct projections from the lateral geniculate nucleus of the thalamus to extrastriate cortical areas such as V4, as suggested by computational simulations [23] or implied by the observation of a parallel, near-simultaneous distribution of input signals in the visual system of other species such as the cat [24]. Thus, while the arrangement of the VCH does not help to predict stimulus processing latencies, the topological scheme does. It also appears to align better than the VCH with features such as the complexity of visual representations in different cortical areas, for instance, V2 and V4, which are more similar in their response properties than expected based on the clear separation of these areas in the VCH [16]. More generally, a topological sequence of converging projections provides a simple model for the increasing receptive field size as well as complexity of visual representations at subsequent processing stages [25,26].

Generalizing the concept of a topological sequence of connections, we wondered how one could decide more formally if brain networks, such as the primate visual cortical network, are organized more sequentially than other comparison networks of the same size. The notion of a sequential organization of brain networks is potentially rooted in the image of the sensory-motor arc, the intuitive incremental processing sequence of sensory inputs eventually resulting in motor actions. However, several known aspects of brain network organization also appear incompatible with a clearly sequential arrangement. First, nodes in brain networks are very widely connected, ruling out a strict serial and sequential chain of signalling in which each node only possesses two connections (i.e. input and output). Moreover, these connections are not just formed with next (spatial or topological) neighbours. Second, shortest paths across brain networks are known to be quite short (e.g. [27]) also indicating the presence of shortcuts across networks, which increase the diversity of processing and reduce the sequential organization.

To assess formally the degree to which brain networks are arranged sequentially, that is, are arranged serially as contrasted to a connectional organization that is parallel or all-to-all, we defined an objective function describing sequential network organization. Basically, a perfect connectional sequence would be an arrangement of nodes A, B, C and D, where A connects to B, B to C, C to D and so on. Naturally, in dense networks, the nodes may also have further connections across the network, so the challenge is to identify the layout that best reflects the serial organization of projections, where the total distance spanned by the projections between connected nodes is minimal. For the formal analysis, the connections need to be embedded in some layout, for instance, simply along a one-dimensional axis. Then the nodes are rearranged until the cost is minimal, which can be achieved through an optimization approach. The minimal cost of the sequential layout of the actual data should also be compared to the layout cost for a network of the same size and density, but different topology, in order to place the organization of the networks on a relative scale.

**Figure 4.**
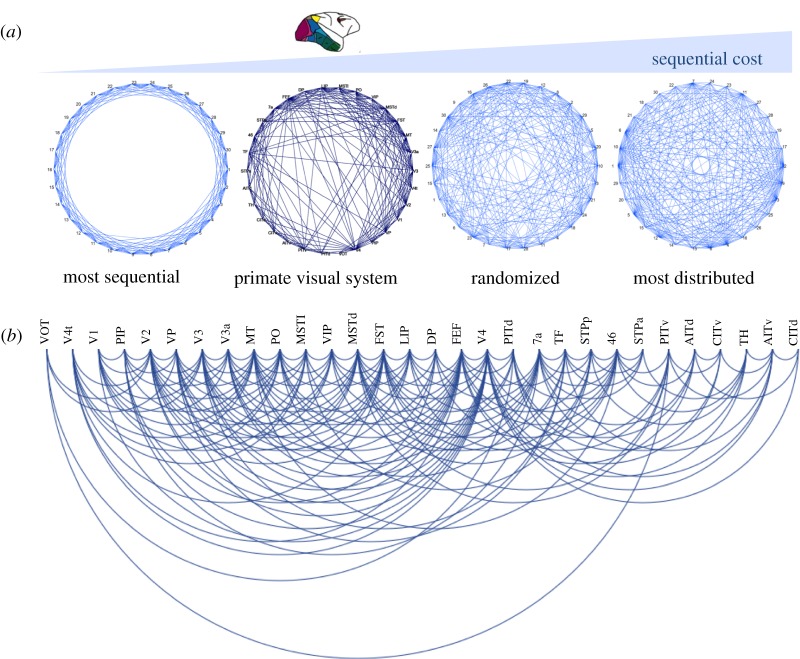
Sequential topology of the primate visual system connectivity versus benchmark networks. (*a*) Minimization of the sequential layout of the primate visual cortical network and comparison networks with the same number of nodes and edges around a circular layout. Areas are placed around a circle in such a way that the total sum of the distances of all connected areas becomes minimal. By the value of this cost function, the organization of the actual network was found to be more sequential than that of randomized networks or other comparison networks, but less sequential than that of strictly sequential networks in which connections are arranged such that they only link immediate neighbours. (*b*) Optimal sequential layout of the primate visual cortical network along a linear one-dimensional axis. The arrangement largely resembles the expected sequence through the system, from primary visual areas (on the left) to higher-order areas (on the right). (Online version in colour.)

An arrangement of this kind for a one-dimensional circular layout is shown in figure 4*a*, as well as for a one-dimensional linear layout in figure 4*b*. The sequential cost of the arrangement of the primate visual cortical network [2] is higher than that of the strictly sequential benchmark networks, but lower than that of other comparison networks, such as randomized or most distributed networks with the same number of nodes and edges. This finding means that visual cortical projections in the primate indeed possess a semi-sequential organization, even if they are not arranged in a strictly sequential way. The sequential arrangement (figure 4*b*) largely resembles the intuitively assumed sequence of the processing of sensory signals in the primate visual system, from primary and adjacent extrastriate areas on the left to dorsal stream and ventral stream areas towards the right. (This interpretation ignores outliers with few documented connections, such as areas VOT and V4t, which are moved to the periphery of the arrangement owing to the sequence optimization process.) The approach demonstrates that presumed aspects of the sequential organization of brain networks can be confirmed and clarified through formal topological analysis. Given the example of the visual system above, the generalization of the topological analysis of projections should also be suitable for explaining signal processing latencies across other networks. More generally, this perspective may be helpful for assessing the communication between and among brain regions [28], and help to identify major paths for communication as well as vulnerabilities and bottlenecks of the sequential organization of connections. A detailed exploration of this concept should be the subject of a separate study.

## 3. Hierarchy as a gradient of features

**Figure 5.**
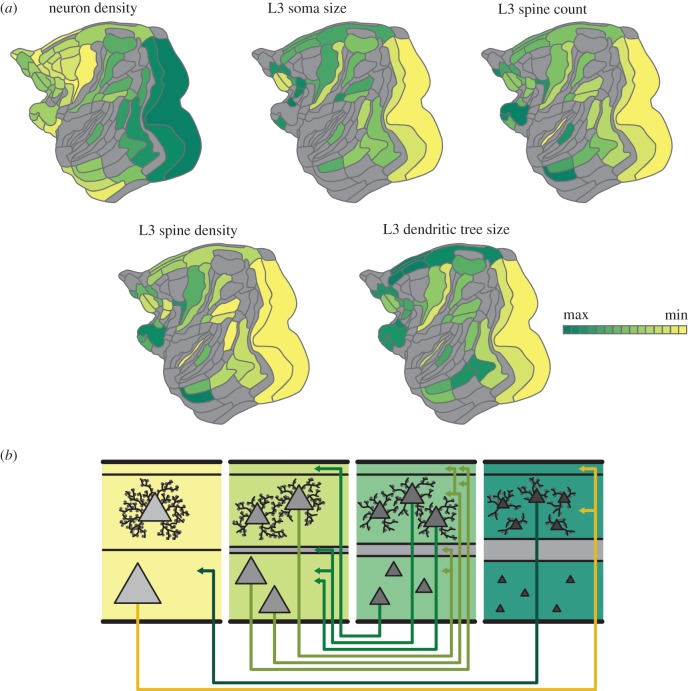
Architectonic gradients of the primate cerebral cortex and their relation to the organization of cortico-cortical connections. Less architectonically differentiated, agranular, cortical areas (yellow) are characterized by lower neuron density and different morphology of layer III pyramidal cells than more strongly differentiated, eulaminate, areas (dark green), with gradual changes across the spectrum. (*a*) Macroscopic and microscopic architectonic features show concerted changes along spatial gradients of the macaque cerebral cortex, indicating a natural axis of cortical organization. In particular, higher neuron density tends to correlate with smaller cross sections of the soma and the dendritic tree as well as with lower total spine count and lower peak spine density. (*b*) Relations of architectonic types with connection features. Connections exist predominantly between areas of similar cortical type; thus, agranular and dysgranular regions (yellow) tend to form more connections with each other than with eulaminate regions (dark green). Moreover, laminar patterns of projection origins are related to differences in architectonic differentiation. Connections between areas of distinct differentiation show a skewed unilaminar projection pattern, with projections originating predominantly in the infragranular or supragranular layers depending on the direction of the projection (agranular to eulaminate projections and eulaminate to agranular projections, respectively), while connections between areas of similar architectonic differentiation show a bilaminar projection origin pattern (connections between middle panels), where the dominating laminar compartment again depends on the connected areas’ relative differentiation. In summary, there are concurrent changes of macro- and microstructural cellular and connectional features across the cortical sheet, forming spatially ordered gradients, confirming and expanding observations from classic neuroanatomy studies (gradation principle of Sanides [29]). Figure adapted from [30].

Another, frequently employed sense of hierarchy is that of a gradient of structural or functional features, that is, an orderly spatial progression of parameters which are changing systematically across the cortical sheet. An example is shown in figure 5*a*, where different macroscopic structural features, such as neuron density, and microscopic features, such as characterizations of layer 3 pyramidal neurons in terms of their size and spine parameters, are shown in the same cortical flat map (adapted from Markov *et al.* [31]). The figure confirms what has been known for a long time (e.g. [32]), that features such as cortical thickness or the neuron density of cortical areas are not distributed uniformly or randomly across the cortical sheet, but are found in systematic variations along spatially organized gradients.

In particular, classic neuroanatomical studies by von Economo and Koskinas demonstrated that cortical areas can be categorized into distinct, ordinal cytoarchitectonic types [32,33]. Moreover, the cytoarchitectonic and myeloarchitectonic inhomogeneity that is observed in the cerebral cortex forms spatially ordered changes, that is, gradients [29]. Generally, such observations suggest that the cerebral cortex is characterized by the so-called gradation principle (*Gradationsprinzip*) [29]. While these spatially ordered changes of cortical features are also frequently called ‘hierarchies’, in the present context the term specifically denotes a succession of spatially ordered changes of a feature, such as cell or spine density or the extension of area-intrinsic projections [34]. Importantly, recent observations [35–40], conjointly with classic studies [29,41], indicate that the spatially ordered changes of cortical features are *concerted*; that is, changes in multiple features, for instance, myeloarchitecture, cell density, spine density, and intrinsic connectivity, occur simultaneously along the same axis across the cortex. For instance, in the case of the structural features displayed in figure 5*a*, overall neuron density of primate cortical areas is anti-correlated with microscopic cellular features, such as the soma cross section of layer III pyramidal cells as well as their spine density and spine count.

It has also been suggested that such architectonic gradients shape basic features of area-to-area cortical connectivity [42]. In particular, in her ‘structural model of connections’ [43], Barbas proposed that laminar terminations and origin patterns of prefrontal cortex are directly linked to the relative differences in the laminar differentiation and organization of cortical areas [44,45]. Indeed, recent work on cat and monkey cortex indicates that cytoarchitecture-based predictive models of laminar origin of connections consistently outperform other predictive models based on features, such as rostro-caudal distance [39,46,47]. Thus, cytoarchitectonic gradients of the cortex, as fundamentally characterized by neuron density, constitute the central axis around which shifts of the laminar origin of connections manifest [40]. Thus, hierarchies based on the laminar origin of connections and hierarchies in the sense of spatially ordered features of cortical organization naturally align (cf. §5).

In summary, a plethora of findings from classic and more recent studies imply that variations of cortical features on multiple levels of cortical organization, such as the genetic, cytological and connectional level, manifest as concerted changes along spatial axes [48]. Such concerted changes are aligned along a common spatial dimension spanning from less to more differentiated parts of the cortex, on average corresponding to a gradient from ‘limbic’ (agranular and dysgranular) cortices to primary, eulaminate cortices (e.g. primary visual cortex); thus, defining a natural axis of variation of cortical organization.

Evidently, the systematic variations of cortical structure and function have ramifications for function. For instance, spine density gradients entail different levels of excitability of the neuronal populations. Indeed, a computational model embodying such density heterogeneity reveals different receptive time windows that correspond to the natural axis of the primate cerebral cortex [49] in line with empirical evidence [50]. In addition, the natural axis of the concerted changes across the cortex entails a sensory to transmodal functional gradient, as classic organizational schemes [3] and more recent observations [51] indicate. Thus, instead of using ‘hierarchy’ to denote the spatially ordered, concerted variation of cortical features, a more neurobiologically concrete term, rooted in a large body of classic and recent studies in different mammalian species, would be a natural gradient or fundamental *axis* of the cerebral cortex.

## 4. Hierarchy as a progression of scales

**Figure 6.**
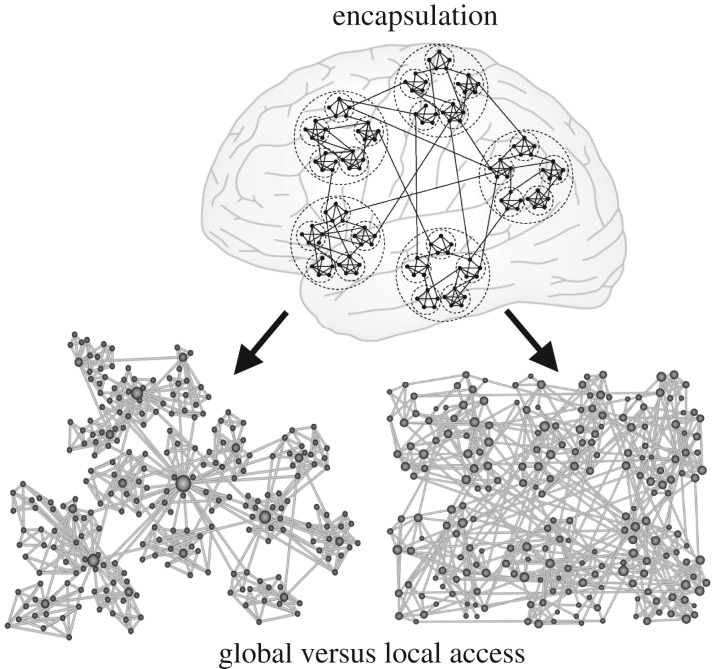
Schematic of hierarchical multi-level modular network organization. This nested module-within-module architecture can comprise diverse types of networks, for example, with (left) or without (right) central hub nodes. The nodes are also differentiated by scales of network access, distinguishing nodes with global access (hub nodes) from local nodes. Adapted from [53,54].

A further interpretation of ‘hierarchy’—which may be more prevalent in the context of network science—is that of a progression of features by different *scales*, not just an orderly sequence or gradient of features as discussed in the preceding sections. In the context of brain networks, where ‘nodes’ abstractly represent neural elements ranging from neurons to whole cortical areas or subcortical nuclei and ‘edges’ represent the structural or functional interactions of these elements [52], such scaling means that smaller network units are contained or encapsulated within larger ones. Several other features may scale in association with this encapsulation of network elements, such as spatial scales or time scales, or a variety of concrete functional features. A straightforward example for such a hierarchical scaling of brain connectivity is a hierarchical modular network (HMN), which is organized in terms of modules of modules of modules (figure 6).

Indeed, it is an intuitive and popular idea that the brain, and in particular the cerebral cortex, is organized into modular networks across many scales, from cellular circuits, cortical columns via nuclei or cortical areas to large-scale units such as the entire visual or sensory-motor cortex. At each level, nodes are more densely wired within than between the modules [55]. While empirical data confirm this modular organization at some scales, for instance, for mesoscopic cortical connections [27], the detailed organization of brain networks across all scales is not yet experimentally accessible. Because of the popularity of the concept of hierarchical modular networks [56], some of its implications are sketched out below.

Concentration of connections within modules allows locally sustained activity, while at the same time preventing global over-excitation of the networks, owing to the low density of inter-modular connections [53]. More generally, hierarchical modularity may be a natural way to balance network segregation and integration. An influential concept in network neuroscience has been that an optimal balance between these aspects reflects the complexity of network organization [57], maximizing the richness—measured by the entropy—of potential functional interactions. The hierarchical, multi-scale organization combines connectional sparsity at the global network level with network integration through connectivity that scales naturally from the local to the global level. Computational studies demonstrate that, both, increasing the number of modules or hierarchical levels increases the stability of self-sustained network activity [58].

At the heart of this observation may be the increased length of cycles by which neuronal nodes in hierarchical modular networks connect back onto themselves. It is apparent that increased network sparsity of large and structured HMNs leads to a general lengthening of cycles. While the average cycle length of dense and small unstructured networks is short, sparser and larger networks display an admixture of long cycles that increases the average cycle length of the network. Simulations with excitable networks demonstrate that activity tends to die out quickly in small and dense networks, as nodes cannot recover their excitability before the next wave of activity arrives. By contrast, networks with a longer average cycle length, which may outlast inhibitory and refractory periods of the nodes and give them time to recover, might be better able to sustain activity patterns. Thus, long cycles in hierarchical modular networks can serve as dynamic reservoirs of neural activity [59]. Computational models also show that structured neural networks such as hierarchical modular networks that combine short with long cycles possess a richer dynamic repertoire combining high and low processing frequencies, associated with short, intra-modular cycles and long, global cycles, respectively [60].

More generally, network segregation and the emergence of long cycles owing to the increase of brain network size may also be relevant for criticality, a key aspect of functional brain dynamics. Criticality, the positioning of a dynamical system at the boundary between order and irregular chaotic dynamic behaviour (or disorder) has for a long time been considered an advantageous configuration of biological systems. Critical points are linked to high adaptability, because small changes in an external control parameter can trigger large rearrangements in the internal system state. There is experimental evidence for such phenomena in the brain [61,62] and, intuitively, the normal working brain should be near the critical transition, because it operates between the extremes of complete order, in a silent brain, and the complete disorder of wildly propagating, unstructured activity. Criticality in the brain is particularly intriguing, because it can enhance the information-processing capabilities of neuronal networks and optimize the dynamic range as well as the input sensitivity of brain networks [63]. However, the apparent operation of the brain in a critical regime is also puzzling, because critical points can typically only be attained through careful fine-tuning or by feedback mechanisms that allow the system to self-stabilize at the critical point. The sparsity of the human brain network with its long cycles suggests an alternative way for generating an extended parameter range of critical behaviour via regional mixtures of sub- and supercritical behaviours, a phenomenon known as Griffiths phases [64]. Signatures of Griffiths phases were found in synthetic hierarchical modular networks as well as examples of neural networks, including the macro-connectome of the human brain inferred from diffusion imaging [65,66].

In summary, the interpretation of ‘hierarchy’ as a scaling of structural and functional properties of the brain appears as a very powerful and general concept that needs to be more fully explored. Several further aspects of the brain show hierarchical scaling, such as the spatial encapsulation of smaller neural elements (e.g. ion channels, spines) in larger ones (i.e. neurons), the hierarchy of time scales and rhythms of the brain [67] as well as the level of access or control of brain networks (local versus global access, cf. figure 6). Finally, such a scaling may also concern the representational level, that is, the chosen ‘aperture’ (scope or focus) of empirical investigations or the complexity of models of neural properties, as in the gradual transition from detailed biophysical models to more abstract representations of neural properties in neural point models or neural masses [68].

## 5. Hierarchy as a sorting of laminar projection patterns

**Figure 7.**
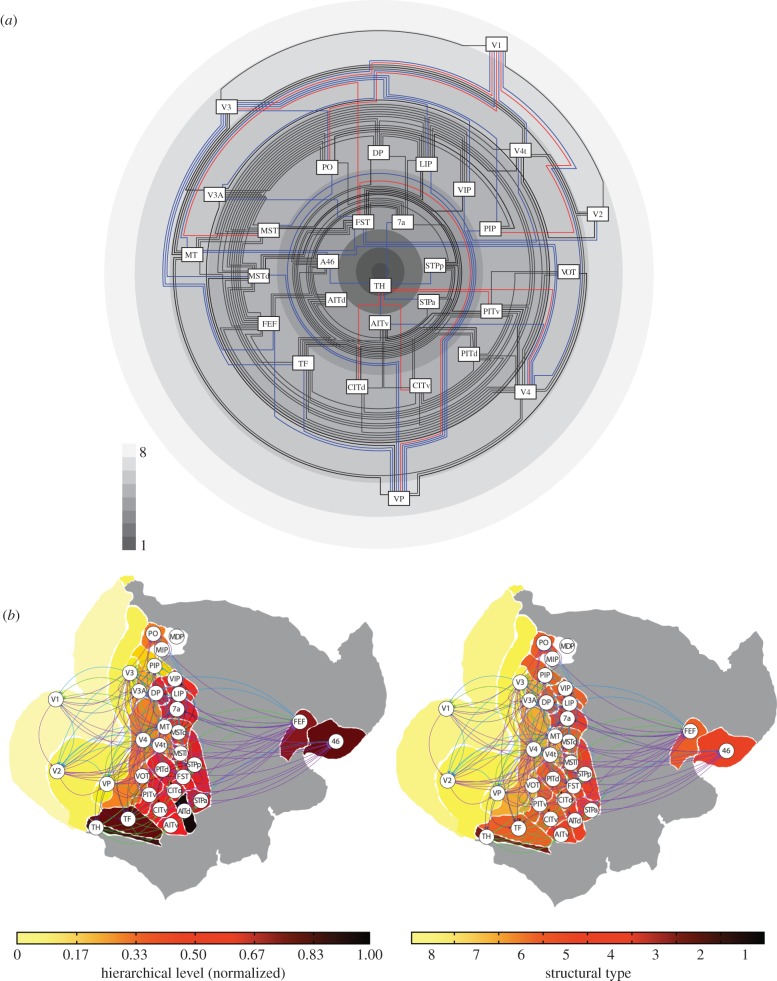
Organization of the connectivity of the primate visual cortical system according to the architectonic type principle [30]. (*a*) Areas are arranged from higher types with dense, well-differentiated layers on the outer rings of the diagram proceeding to lower type areas on the inner rings of the scheme. Types are indicated by the shading of the rings, with lighter shading for higher and darker shading for lower types, as shown by the grey level scale. Connections, based on [2], between areas of the same or neighbouring types are drawn in black, between areas separated by two types in blue, and projections between areas separated by more than two types are shown in red. The predominance of black projections indicates the consistency of the structural model. (*b*) Comparison between average cortical hierarchy and structural types of the primate visual system. Left, diagram adapted from [12]; right, structural types. While there are small apparent differences between these figures, the overall picture is quite similar. This observation is owing to the fact that the architectonic type principle implies that structural type differences are correlated with laminar projection patterns, and, thus, the structural type scheme underlies the hierarchical arrangement of areas resulting from the sorting of the oriented projections. Figure adapted from [69].

Let us return to the initial example of the VCH (§1b). It turns out that the progression of amazingly regular projection patterns of visual cortical areas in the primate brain (figure 1*a*) can be related to the interpretation of ‘hierarchy’ as a gradient of structural features, as discussed above (§3). In particular, laminar projection patterns may be explained from the close relationship between the relative architectonic type of projection source and target and the laminar projection origins and terminations [44,45,69]. As there are systematic, graded variations of laminar architecture across the cortical sheet, the projection patterns also vary systematically along the structural gradients (figure 7). In this perspective, the cortical architectonic gradients determine the laminar patterns, which in turn specify the VCH. It is, therefore, no surprise that gradients of cortical types and sortings of laminar patterns appear highly similar, but anti-correlated (high-type areas are sources of mostly ‘forward’ projections, which places them at the origin of laminar hierarchies, whereas low-type areas are sources of mostly ‘backward’ projections which puts them at the end point of laminar hierarchies; figure 7*b*). The difference is that cortical types are well-defined biological entities with a direct structural and functional interpretation (in terms of neuron density, spine count, branching of intrinsic connections, neurotransmitter receptor profiles, excitability, and so on), whereas the levels of the VCH are constructs derived indirectly from an (indeterminate) sorting process with no such clear interpretation. The VCH can, therefore, be considered as an epiphenomenon of the gradients of cortical structure. This conclusion does not contradict the fact that the VCH may be correlated with structural or functional features such as excitability of cortical areas [49]; however, a explanation for these features should not be sought in the VCH, but the underlying structural gradients of the cortex. For instance, excitability may be explained more fundamentally by variations of neuronal density versus synaptic density. More generally, as the basic presence or absence of connections is also linked to the architectonic similarity of primate cortical areas [30,40,43,69], there exists a natural relationship between the architectonic gradients of the cortex described in §3 and the sequential organization of cortical connections outlined in §2 above.

To wrap up, while the restating of the traditional picture of the VCH in terms of cyto-architectonic gradients may be a challenging change of perspective, a clearer focus on the underlying factors of this arrangement will be helpful for a deeper, biologically meaningful understanding of projection patterns in the context of cortical architecture.

## 6. Conclusion

While the term ‘hierarchy’ is frequently invoked in systems and network neuroscience, it can stand for very different concepts, ranging from the sorting of connections by their laminar projection patterns to the scaling of diverse connectional and functional features. Such a diversity of uses of the term is perfectly acceptable, of course, as long as the intended meaning of ‘hierarchy’ in the context of a particular concept is made clear. Otherwise the use of the term ‘hierarchy’ becomes meaningless or, worse, misleading.

**Figure 8.**
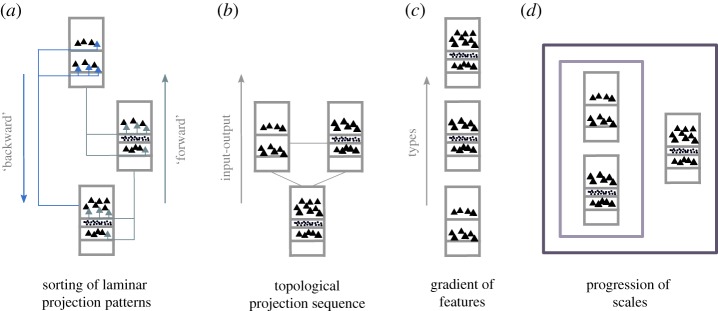
Overview of different concepts of ‘hierarchy’ in cortical brain networks as reviewed in the present paper. Depending on the chosen ‘hierarchy’ concept, the arrangement of the areas may vary substantially, also leading to different expectations of their functional properties. This overview is not exhaustive, and further notions of ‘hierarchy’ may be identified in the literature. (*a*) Sorting of areas by their ‘forward’ and ‘backward’ projections as classified from the laminar patterns of projection origins and terminations. (*b*) Arrangement of areas by the topological sequence of their connections, according to shortest paths from inputs at the bottom to outputs on the top. (*c*) Sorting of areas by feature gradients, for instance of cortical types or cellular density increasing from bottom to top. (*d*) Arrangement of areas by a progression of scales. Smaller neural systems are encapsulated in larger ones. For instance, laminar compartments are contained in cortical areas, which are in turn grouped into increasingly larger systems, such as the ventral and dorsal ‘streams’ of the primate visual system [73], by the arrangement of their connections and their functional properties. (Online version in colour.)

In the present review, we revisited aspects of ‘hierarchy’ that more concretely relate to repeated patterns of laminar projection origins and terminations, the sequential topology of projections, gradients of structural and functional features across the cortex, as well as the scaling and encapsulation of features of the connectional topology (figure 8). As particularly demonstrated for the example of the non-human primate cortical visual system, these diverse ‘hierarchy’ concepts lead to different interpretations of the empirical data, with diverging functional implications. Whereas the interpretation of ‘hierarchy’ as a sequential progression of projections in the primate cortex may help to explain latencies and increasing complexity of representations, the interpretation as cortical gradients may be helpful for explaining excitability and time scales of processing, whereas the interpretation as a scaling of features may help to explain self-sustained activity and critical phase transitions. Consequently, the use of more concrete terms that are tailored to the specific contexts should help to disambiguate the polysemy and ambiguity of the use of the term ‘hierarchy’ and bring us closer to the core organizational principles of the cerebral cortex.

Here, we focused on four different uses, and thus, meanings of the term ‘hierarchy’ (figure 8). This list should not be considered exhaustive. In fact, we expect that further uses and meanings of the term can be discerned from the neuroscience literature. For instance, some studies and models treat hierarchy in terms of control between different brain structures. Specifically, it was postulated that the prefrontal cortex is organized in a hierarchical manner, with the frontal most part positioned at the top of this hierarchy [70]. In this interpretation, the top of the hierarchy is adequately equipped at the connectional level to exert strong influence on other regions, owing to the larger number of outgoing connections to the remainder of prefrontal areas. Thus, on a connectional basis, another potential use and meaning of the term ‘hierarchy’ can be employed, in terms of hierarchy as control or dominance.^3^ Importantly, such a definition can also be formalized in graph theory terms and be subsequently examined in empirical and synthetic neuronal networks [71]. In summary, further meanings of the term ‘hierarchy’ may expand and enrich the existing list and we hope that this endeavour will further enhance the conceptual clarity that is needed for proper dissemination of ideas, results, models and theories among neuroscientists.

While we have emphasized the context-specific interpretations of the different aspects of ‘hierarchy’, there also exist multiple relations between them. This fact was most clearly demonstrated by the close relationship between gradients of cortical types and the progression of laminar projection patterns, as expressed in the architectonic type principle (§5). Ultimately, all the different aspects of ‘hierarchy’ are integrated through the embedding of connections in the spatial and topological architecture of the brain [30,72], where they underlie multiple interwoven structural and functional features that give rise to the intricate activity patterns and functions of the nervous system.

## Endnotes

1 In this paper, we use ‘hierarchy’ when we mean the vague, intuitive use of the term and otherwise define hierarchy concretely in specific contexts.

2 We have put the labels of projection direction into quotation marks, because the general association between the assumed direction and the laminar patterns of cortico-cortical projection is not straightforward. While the relationship appears intuitively clear for projections underlying the initial processing steps of sensory signals (figure 1*c*), it is far less clear for projections further removed from the sensory-motor periphery of the brain—which way is ‘forward’ in the prefrontal cortex?

3 In fact, this meaning aligns with the use of ‘hierarchy’ in common language in the sense of a system of successive ranks, as indicated by the Oxford English Dictionary (https://oed.com/viewdictionaryentry/Entry/86792).

## Data accessibility

This article has no additional data.
